# Terrestrial locomotion of the Svalbard rock ptarmigan: comparing field and laboratory treadmill studies

**DOI:** 10.1038/s41598-019-47989-6

**Published:** 2019-08-07

**Authors:** Andres C. Marmol-Guijarro, Robert L. Nudds, John C. Marrin, Lars P. Folkow, Jonathan R. Codd

**Affiliations:** 1 Faculty of Biology, Medicine & Health, University of Manchester, Manchester, UK; 2 Faculty of Biological Science, University of Leeds, Leeds, UK; 3 Department of Arctic and Marine Biology, University of Tromsø – the Arctic University of Norway, Tromsø, Norway

**Keywords:** Climate-change ecology, Biomechanics

## Abstract

Research into the terrestrial locomotion of birds is often based upon laboratory treadmill experiments. However, it is unclear how transposable these results are for birds moving in the wild. Here, using video recordings, we compared the kinematics of locomotion (stride frequency, stride length, stance phase, swing phase, duty factor) and speed range of Svalbard rock ptarmigan (*Lagopus muta hyperborea*) under field and laboratory treadmill conditions. Our findings indicate that the kinematics of walking and aerial running are conserved when moving on the treadmill and in the field. Differences, however, were found when grounded running under the two conditions, linked to substrate. Substrate effects were confirmed by analysing trials only moving over very hard snow. In line with laboratory treadmill energetic predictions, wild ptarmigan have a preferred speed during walking and to a lesser extent when aerial running but not when moving with a grounded running gait. The birds were also capable of a higher top speed in the field than that observed during treadmill studies. Our findings demonstrate that laboratory treadmill research provides meaningful information relevant to wild birds while highlighting the importance of understanding the substrate the animals are moving over.

## Introduction

Animals may be defined by the way they move around and are often capable of multiple forms of locomotion^1^. For example, although most birds can fly, many species during key times of the year, and for such vital processes such as feeding and mating, are dependent on terrestrial locomotion. Research into avian terrestrial locomotion has tended to focus on locomotor energetics and kinematics from treadmill-based studies (see for example^2–13^). While the treadmill provides a uniform, very hard, rubbery and grippy surface, real world substrates can be anything from grass, mud, loose or firm rocks, snow or ice and combinations thereof. There is comparatively little data from wild free-ranging animals; meaning that information such as speed ranges and gait classifications have yet to be collected under natural conditions^7^. While treadmill studies have facilitated great insight into animal locomotion, they are conducted under idealised conditions^14,15^. These basic tenants of experimental research allow specific parameters of interest to be manipulated without additional factor(s) confounding results. However, it also means the relevance of these results for an animal moving through a constantly changing landscape needs to be established.

Aside from temperature, wind and light, perhaps the principle difference between laboratory treadmill and field conditions is substrate, which is known to affect locomotion. For example, previous work has shown that moving on a treadmill, as opposed to a natural substrate, can affect the ground reaction forces, causing a reduction in the extension and flexion moments of the foot joint in humans^16^. For humans and rats moving on a treadmill there is also an increase in stride frequency concomitant with a decrease in stride length, compared to moving over natural substrates^16,17^. Substrate differences can also require adjustments in the neuromuscular control of locomotion in order to maintain stability^18–22^. Interestingly, locomotion over snowy ground has often been chosen to examine the effect of substrate on locomotion. Moving over snow also affects the locomotor behaviour of animals, in terms of the pathway taken over the ground and the speed at which an animal moves^23–27^. Despite the influence of different conditions, a key assumption in all studies into the evolutionary significance of locomotor adaptations is that laboratory treadmill studies reflect what the animals do under natural conditions^28^.

Research into the congruence between field and laboratory treadmill locomotion data has largely focussed on the effects of substrate on energy expenditure during locomotion in humans, and has found conflicting results. Some studies note differences in kinematics^16,29–35^ and energetics^36^ while others have found no difference in either of these^37^. For mammals other than humans, the relatively few studies have focused on Artiodactyls, and have demonstrated that elk and mule deer moving in soft deep snow experience an increase in energy expenditure^38,39^. In birds there are even fewer studies. Recent work looking at gait transitions in paddock-housed ostriches indicated that the preferred walking speed of these birds was around that predicted to minimise energy expenditure^14^. Preliminary attempts to analyse substrate effects on Svalbard ptarmigan locomotion^40^ focused only on grass rather than the natural snowy substrate the birds move over in the wild and did not distinguish between the sexes. Aside from these studies, comparative real-world data from birds moving over natural substrates are lacking. Without this research it is difficult to understand how factors that affect locomotion relate to fitness and therefore place any experimental laboratory treadmill data into an evolutionary context^7^. These data are important because without them it is impossible to determine, for example, what the potential impact might be of environmental change.

Here we examined the locomotor kinematics and self-selecting speed of free-ranging wild, male Svalbard rock ptarmigan (*Lagopus muta hyperborea*) on Spitzbergen, Svalbard. The birds were moving over natural snowy substrates and were compared to existing laboratory treadmill data on the energetics and kinematics of their locomotion^7,41^. We aim to determine if we can reliably extrapolate from laboratory treadmill data to the field, using the Svalbard ptarmigan as a model species.

## Materials and Methods

### Study species and data collection

We recorded videos of terrestrial locomotion from wild, free-ranging male *L. muta hyperborea* (n = 91) in the Adventdalen valley and adjacent side valleys on Spitzbergen, Svalbard (78°13′18″N, 15°38′30″E), from 22^nd^ April to 4^th^ May 2017 and the 21^th^ April to 7^th^ May 2018. Svalbard ptarmigan were selected for these studies as they are one of the few avian species where a comprehensive laboratory treadmill dataset exists on the kinematics and energetics of their locomotion^7^. During terrestrial locomotion Svalbard ptarmigan move faster by either changing the number of strides they take in a given time or by increasing the length of each stride, or both. These birds are ideal for locomotor studies as they can make use of up to three terrestrial gaits: walking (where one foot is in contact with the ground at all times), aerial running (where both feet are off the ground) and grounded running (a transitional gait with intermediate characteristics of walking and aerial running). During spring the ground is snow covered, the midnight sun was already present and birds were at their summer weight^42^. Only males, identified from their calls and secondary sexual characteristics (red supraorbital combs and eye-stripe) were used to facilitate comparison with existing laboratory treadmill data^7,41^. Where possible each bird was filmed moving at low and high speed and a total of 165 videos were analysed. Snowmobiles were used to cover the ground between sites. Once a bird was identified it was filmed from a fixed distance moving across level ground at either 25 frames per second (fps) using a SONY® Handycam HDR-XR250 (SONY® Corporation, Japan) during the 2017 season, or at 100 fps using a SONY® Cyber-shot RX10-III camera (SONY® Corporation, Japan) during the 2018 season. While filming the camera was maintained in a fixed position at the same height and parallel to the moving bird. After the bird had been filmed a 1 metre scale bar was then placed along the track way of the animal to calculate speed (*U*). Stride frequency (*f*_stride_) and stride length (*l*_stride_) were calculated for each bird as the average of 3–5 complete strides during which the birds was neither accelerating or decelerating. *f*_stride_ was obtained by dividing the number of strides by the duration of the video clip and *l*_stride_ was calculated as *U* divided by *f*_stride_. Data from the 100fps videos were used to measure stance (*t*_stance_) and swing (*t*_swing_) duration, and duty factor (DF), apart from *l*_stride_ and *f*_stride_. To reduce pseudo replication of data, bird locations were GPS marked and those locations were used only once. Data collection techniques used in the field were refined from preliminary attempts at comparing treadmill locomotion of captive Svalbard rock ptarmigan to birds moving within an outdoor race over grass^40^.

Previous laboratory treadmill-based studies by our group identified that the Svalbard rock ptarmigan use three different terrestrial gaits identified by the phase relationship between *E*_hk_ (horizontal kinetic energy vector) and *E*_p_ + *E*_vk_ (the sum of the potential and vertical kinetic energy vectors) of the centre of mass (CoM)^7^. During walking *E*_hk_ and *E*_p_ + *E*_vk_ fluctuate out of phase, whilst during grounded and aerial running *E*_hk_ and *E*_p_ + *E*_vk_ are in phase^43^. To confirm gaits across the speed range for wild ptarmigans, we tracked the movement of the CoM of birds to determine *E*_hk_ and *E*_p_ + *E*_vk_ from the 100 fps recordings. The location of the CoM was identified relative to known morphological points; by using the points to build a polygon and then using it to estimate the centre of mass. Points used were either the proximal end of the neck or the eyeball of the bird, the proximal end of the tail, and the sternum. Mean body mass estimates were taken from literature values^41,44^. To further test the influence of substrate on our results we re-ran the analysis having excluded all trials other than those moving over very hard snow. This was done to allow a close to like-for-like substrate comparison between the animals moving in the wild and on a treadmill in the laboratory, with its uniform firm surface. Video analyses were conducted using Tracker® v. 5.0.5 (Open Source Physics). Substrate classification over which the birds were moving (electronic Supplementary Material, ESM, Table S1) was conducted during locomotor trials was based the hardness of deposited snow^45^.

Experimental procedures and methods were carried out under ethical approval from the University of Manchester Ethics Committee in accordance with the Animal (Scientific Procedures) Act 1986, covered by Home Office project licence (40/3549).

### Statistical analyses

To check for potential differences in *l*_stride_ and *ƒ*_stride_ between years we performed ANCOVA’s for each of the parameters using *U* as a co-variate. Walking, grounded and aerial running are distinct gaits and were therefore analysed separately in all analyses. All kinematic parameters within each gait were analysed using linear regression. *t*_stance_ and *t*_swing_ data (and *U* for these two parameters) for locomotion in the wild and on a laboratory treadmill were linearized using a log_10_ transformation prior to analyses. To facilitate comparison between our results for wild and laboratory treadmill datasets, we reanalysed the laboratory treadmill data from our group using linear regression within each gait. Shapiro- Wilks tests were then performed on the residuals of each linear model to ensure the data were normally distributed. Once the linear models were derived two-tailed Z-tests were performed, in order to identify any differences in the slopes of each kinematic parameter between the data from wild ptarmigans and that of the existing laboratory treadmill dataset. Z-tests were used, as they are robust to violations of the assumption of equal variances for two samples. Only the intercepts for the walking gaits were compared, because doing the same for grounded running and aerial running would be extrapolating the lines of best fit too far beyond the data range rendering their estimates unreliable. All statistical analyses were conducted in R v.3.4.3^46^ and results are summarized in Tables 1 and S2.

**Table 1:** Results of the linear regressions of each kinematics parameter against *U* for each gait and the *z*-test comparisons of the slope and intercept coefficients.

Gait	Parameter	model		Slope		Intercept	
		Field	Laboratory treadmill	*z*	*p-*value	*z*	*p-*value
Walk	*l*_stride_	0.142 + 0.204 *U* (*t* = 9.287, *r*^2^ = 0.65, *n* = 48, *p* < 0.001)	0.102 + 0.246 *U* (*t* = 5.319, *r*^2^ = 0.96, *n* = 3, *p* < 0.001)	−0.837	0.401	1.362	0.174
	*ƒ*_stride_	1.022 + 2.051 *U* (*t* = 10.08, *r*^2^ = 0.69, *n* = 48, *p* < 0.001)	1.043 + 2.130 (*t* = 36.28, *r*^2^ = 0.99, *n* = 3, *p* < 0.001)	−0.372	0.711	−0.149	0.881
	*t*_stance_	−0.679–0.720 log_10_ *U* (*t* = −10.97, *r*^2^ = 0.83, *n* = 26, *p* < 0.001)	−0.646–0.623 log_10_ *U* (*t* = −7.368, *r*^2^ = 0.98, *n* = 3, *p* = 0.086)	−1.178	0.238	−1.217	0.222
	*t*_swing_	−0.879–0.184 log_10_ *U* (*t* = −2.30, *r*^2^ = 0.18, *n* = 26, *p* = 0.031)	−0.838–0.012 log_10_ *U* (*t* = −0.20, *r*^2^ = 0.03, *n* = 3, *p* = 0.88)	−1.688	0.091	−1.172	0.242
	DF	0.814–0.230 *U* (*t* = −6.21, *r*^2^ = 0.62, *n* = 25, *p* < 0.001)	0.858–0.281 *U* (*t* = −6.72, *r*^2^ = 0.98, *n* = 3, *p* = 0.094)	0.930	0.352	−1.390	0.165
Grounded running	*l*_stride_	0.260 + 0.088 *U* (*t* = 3.74, *r*^2^ = 0.21, *n* = 56, *p* < 0.001)	0.194 + 0.158 *U* (*t* = 7.934, *r*^2^ = 0.95, *n* = 5, *p* < 0.01)	−2.275	< 0.05	—	—
	*ƒ*_stride_	0.979 + 1.925 *U* (*t* = 9.55, *r*^2^ = 0.63, *n* = 56, *p* < 0.001)	1.621 + 1.258 *U* (*t* = 5.728, *r*^2^ = 0.92, *n* = 5, *p* < 0.05)	2.238	< 0.05	—	—
	*t*_stance_	−0.707–1.021log_10_ *U* (*t* = −9.91, *r*^2^ = 0.78, *n* = 30, *p* < 0.001)	−0.656–0.955 log_10_ *U* (*t* = −44.49, *r*^2^ = 0.99, *n* = 5, *p* < 0.001)	−0.845	0.593	—	—
	*t*_swing_	−0.833–0.392 log_10_ *U* (*t* = −3.53, *r*^2^ = 0.31, *n* = 30, *p* < 0.01)	−0.796 + 0.00 log_10_ *U* (*t* = 0, *r*^2^ = 0.51, *n* = 5, *p* = 0.167)	−3.529	<0.001	—	—
	DF	0.689–0.122 *U* (*t* = −4.47, *r*^2^ = 0.42, *n* = 30, *p* < 0.001)	0.734–0.164 *U* (*t* = −18.18, *r*^2^ = 0.99, *n* = 5, *p* < 0.001)	1.479	0.139	—	—
Aerial Running	*l*_stride_	0.144 + 0.162 *U* (*t* = 11.54, *r*^2^ = 0.69, *n* = 61, *p* < 0.001)	0.247 + 0.131 *U* (*t* = 2.484, *r*^2^ = 0.75, *n* = 4, *p* = 0.131)	0.552	0.582	—	—
	*ƒ*_stride_	2.947 + 0.665 *U* (*t* = 5.27, *r*^2^ = 0.32, *n* = 61, *p* < 0.001)	1.788 + 1.078 *U* (*t* = 3.12, *r*^2^ = 0.82, *n* = 4, *p* = 0.089)	−1.122	0.263	—	—
	*t*_stance_	−0.83–0.469 log_10_ *U* (*t* = −3.41, *r*^2^ = 0.28, *n* = 32, *p* < 0.01)	−0.698–0.723 log_10_ *U* (*t* = −3.32, *r*^2^ = 0.85, *n* = 4, *p* = 0.08)	0.983	0.327	—	—
	*t*_swing_	−0.855–0.136 log_10_ *U* (*t* = −1.20, *r*^2^ = 0.04, *n* = 32, *p* = 0.24)	−0.722–0.311 log_10_ *U* (*t* = −1.71, *r*^2^ = 0.59, *n* = 4, *p* = 0.23)	−0.636	0.522	—	—
	DF	0.54–0.042 *U* (*t* = −2.084, *r*^2^ = 0.13, *n* = 32, *p* = 0.045)	0.621–0.095 *U* (*t* = −1.992, *r*^2^ = 0.67, *n* = 4, *p* = 0.185)	1.02	0.308	—	—

The lines of best fit are also given. Only the intercepts for the walking gaits were compared, because comparison for grounded running and aerial running would require extrapolating the lines of best fit too far beyond the data range rendering their estimates unreliable. Statistical significance is set as p < 0.05.

### Ethics

This project was conducted under ethical approval from the University of Manchester Animal Ethics Committee and a permit from the Governor of Svalbard Research in Svalbard (RiS Project No 10790).

## Results

### Gait analysis

**Figure 1:**
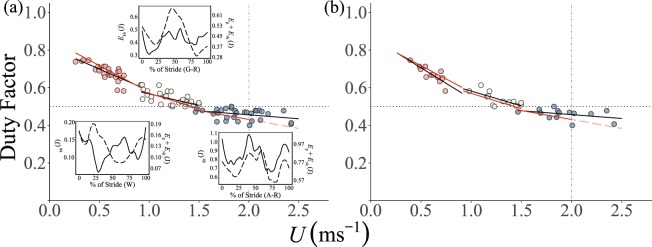
Duty Factor (DF) plotted against speed (*U*) for walking (red), grounded running (white) and aerial running (blue) gaits. (**a**) Includes data points for trials over all snow conditions and (**b**) includes data points for ptarmigan exclusively moving over a very hard snow surface only. Inlay figures in (**a**) represent the horizontal kinetic energy E_kh_(J), and potential plus vertical, E_p_ + E_vk_ (J) energy plots of fluctuations of in the Centre of Mass (CoM) for each gait from one bird; walking (W), grounded running (G–R), aerial running (A–R). On the inlay figures the solid black line within each box are represent the kinetic energy, whereas the dashed black line represents the potential gravitational energy fluctuations. In the main figure the lines of best fit describing the linear regression for wild ptarmigans freely moving in the field are shown in black. The solid red lines represent the lines of best fit for the laboratory treadmill data. The vertical dashed line denotes the maximum sustainable speed from the treadmill data^7^. To the right of the vertical line at 2.0 ms^−1^, the red line becomes dashed to denote projected speed beyond that sustainable in the laboratory. The horizontal dotted line represents the threshold duty factor of 0.5.

No significant differences were found for *l*_stride_ and *ƒ*_stride_ when comparing data across 2017 and 2018 (Table S3), allowing these datasets to be combined. Walking, grounded and aerial running gaits were confirmed from field data. When the fluctuations of *E*_hk_ and *E*_vh_ + *E*_p_ were out of phase the birds were walking and suggested a pendular mechanism of energy recovery^43^, that extended from 0.26 ms^−1^ to 0.91 ms^−1^ (Fig. 1a). Grounded and aerial running gaits were identified from 0.92 ms^−1^ to 2.76 ms^−1^, where the fluctuations between *E*_hk_ and *E*_vh_ + *E*_p_ were synchronized and energy recoveries occur by means other than kinetic energy to gravitational energy transfer^43^. DF was then used to separate grounded running (DF > 0.5) and aerial running (DF ≤ 0.5). The shift between these two gaits occurred at slightly lower speeds than previously suggested^7^ and overlapped between 1.46 ms^−1^ – lowest aerial running speed – and 1.50 ms^−1^ – highest grounded running speed (Fig. 1a).

### Walking gait kinematics

**Figure 2:**
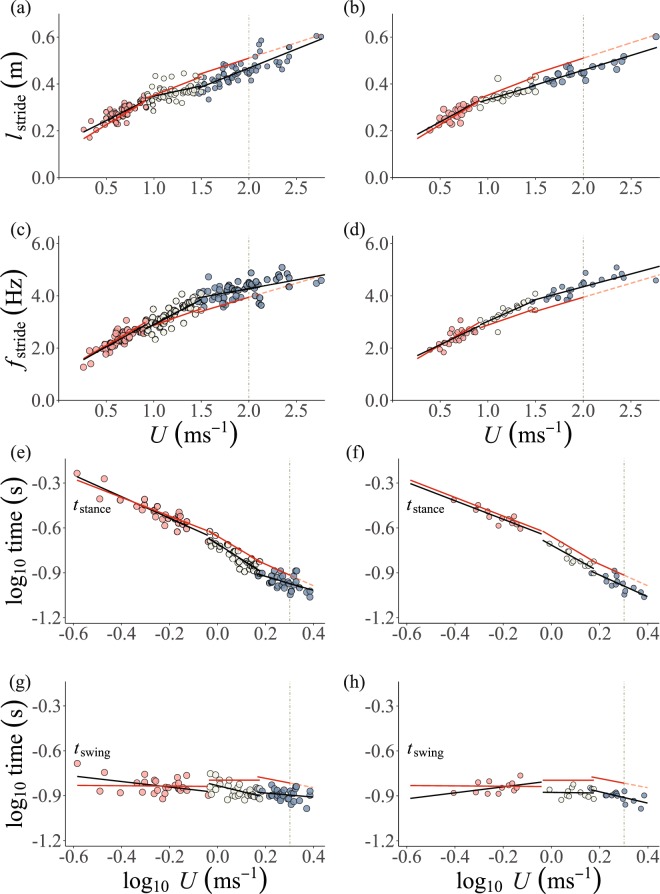
Kinematic parameters plotted against *U* for each gait - (**a**,**b**) *l*_stride_ against *U*; (**c**,**d**) *ƒ*_stride_ against *U*; (**e**,**f**) log_10_ *t*_stance_ against log_10_ *U*; and (**g,h**) log_10_ *t*_swing_ against Log_10_ *U*. The left panels (a,c,e,g) represent data points for the trials over all snow conditions. The right panels represent data points for ptarmigan exclusively moving over very hard surfaces to facilitate like-for like substrate comparisons between field and laboratory treadmill datasets. Walking, ground running and aerial running gaits are denoted by the red, white and blue circles, respectively. The lines of best fit describing the linear regression for wild ptarmigans are shown in black. The red lines represent the lines of best fit for the laboratory treadmill data. The vertical dashed line denotes the maximum sustainable speed recorded in the laboratory treadmill experiments^7^. To the right of the vertical line at 2.0 ms^−1^, the red line becomes dashed to denote projected speed beyond that sustainable in the laboratory.

*l*_stride_ and *ƒ*_stride_ increased linearly with *U* (Fig. 2a,c, Table 1) for both the field and laboratory treadmill derived measurements. Log_10_ *t*_stance_ and log_10_ *t*_swing_ decreased linearly with log_10_ *U* and DF with *U* for the field data (Figs 1a, 2e,g and Table 1). Similar trends are seen in the laboratory treadmill data, although the decrease in *t*_swing_, *t*_stance_ and DF with *U* was not supported statistically (Figs 1a, 2e,g and Table 1). None of the relationships between the kinematics parameters and *U* differed (neither intercepts nor slopes) between the field and laboratory treadmill data (Table 1). Birds used walking gaits in 48 trials, from which 25 (52%) were over very hard snow.

### Grounded running gait kinematics

*l*_stride_ and *ƒ*_stride_ increased linearly with *U* for both field and laboratory treadmill measurements (Fig. 2a,c, Table 1). The incremental increase in *l*_stride_ with *U*, however, was greater in the laboratory treadmill data than in the field data (Table 1). In contrast, the incremental increase in *ƒ*_stride_, was less in the laboratory treadmill data than in the field data. Log_10_ *t*_stance_ decreased linearly with log_10_ *U* and at a similar rate in both data sets (Fig. 2e, Table 1). log_10_ *t*_swing_ decreased linearly with log_10_ *U* in the field data, but was not affected by *U* in the laboratory treadmill data (Fig. 2g, Table 1). The linear decrease in DF with increasing *U* was similar in both field and laboratory treadmill data (Fig. 1a, Table 1). A grounded running gait was used in 56 trials, from which 23 (41%) were over very hard snow

### Aerial running gait kinematics

*l*_stride_ and *ƒ*_stride_ increased with *U* in the field and laboratory treadmill data although these trends were not statistically significant in the latter (Fig. 1a). Log_10_ *t*_stance_ decreased linearly with log_10_ *U* in the field and a similar trend (*p* = 0.08) was seen in the laboratory treadmill data sets (Fig. 2e). Log_10_ *t*_swing_ did not change with log_10_ *U* in either field or laboratory treadmill data sets (Fig. 2g). DF decreased linearly with *U*. For all the kinematic parameters, the relationship with *U* did not differ between field and laboratory treadmill data (Fig. 1a, Table 1). Aerial running was used in 61 trials, of which 24 (39%) were over very hard snow. There were no aerial running trials over soft or medium snow.

### Like for like comparison of kinematics over very hard snow

When only data for birds moving over very hard snow were analysed no differences were detected in the laboratory treadmill and field data kinematics for each specific gait (Figs 1b, 2b,d,f,h and Table S2).

### Self-selected speeds

Counts (binned into arbitrary 0.07 ms^−1^ increments) were used to determine the frequency with which each speed was selected and a density bandwidth plot (right axis) was added in order to visualize the speed distribution for ptarmigan within each gait (Fig. S1). The density bandwidth was automatically estimated using the ggplot2 package for R. Ptarmigan in the field used a range of *U* from 0.26–2.76 ms^−1^. Probability density estimations (Fig. S1), however, suggest that walking at around 0.7 ms^−1^ and to a lesser extent aerial running around 1.7–1.85 ms^−1^ was preferred. Generally, the birds infrequently moved at very slow (0.1–0.4 ms^−1^) and very high (>2.1 ms^−1^) speeds. There was no obvious preferred speed within the grounded running gait (Fig. S1).

## Discussion

It is imperative to our understanding of animal locomotion that we can be confident that locomotion data collected from laboratory treadmill studies is representative of natural movement in the field^40^. These results provide new insight into the congruence between field and laboratory treadmill data. Our findings show that the kinematics of locomotion in the Svalbard ptarmigan when walking and aerial running are conserved across laboratory treadmill and field datasets where the birds were moving over snow. These findings intuitively make sense as both walking and aerial running have clear evolutionary relevance. Birds use a walking gait when foraging for immobile food objects and general exploration^7^, while aerial running functions in predator escape and facilitates the economic movement over large distances^7,10,47,48^. The birds in the current study utilised walking gaits over a range of substrates from soft, powdered snow to mixed and harder snow as they were commuting between feeding sites, most often tending to use the relatively faster walking speeds which are the most energetically efficient^7^. Optimal foraging theory suggests that foraging decisions (like how and where to move) are made to maximise fitness-related currencies based on combinations of the energy and time to be expended^49,50^. In other words natural selection should favour animals that forage the most efficiently^51^.

Svalbard ptarmigan feed by pecking at the ground, scratching away the snow with their feet to uncover vegetation as they move around, primarily selecting feeding sites that are loosely covered in snow. Selecting a relatively fast walking gait when foraging is the most efficient means for the Svalbard ptarmigan to commute between sites while still being able to identify and access food sites, as found in other species of birds^52^. Moving slowly can also negate the negative effects of moving through a substrate that might otherwise result in an increase in the energetic cost of movement^53^. Conversely, we found the birds used aerial running gaits exclusively on firmer snow suggesting that the bouncing mechanism, linked to elastic energy recovery during the stance phase when running^54,55^, only functions when moving over firm ground. Other animals, for example many mesopredators, in snowy conditions also demonstrate a preference for moving over shallow compressed snow either to minimize energy expenditure^23–27^ or simply to travel faster^26^. A softer substrate would absorb some or (all) of the kinetic and potential energy during the stance phase reducing the elastic energy available for the next stride^55^. Other links between the type of substrate and locomotor gait have previously been noted with slow speed walking linked to softer snow and higher speed running on hard snow in humans^56^. The selection of gaits depending on substrate correlates with increases in energy expenditure which relate to the depth of footprints or trackways^23–27,38,56–58^.

Interestingly, differences in kinematics were found when the birds were moving with a grounded running gait in the field compared to the laboratory treadmill data, when all snow types were considered. Ptarmigan moving with a grounded running gait in the wild took faster, smaller steps for the same speed range as laboratory treadmill studies. Grounded running is an intriguing gait as it links duty factors over 0.5 with running-like energy fluctuations in the centre of mass^59^. Grounded running is associated with more compliant limbs and improves visual stability through better control of head movements^47^ and reduces the mechanical work of the bouncing non-locomotor body tissues^21^. It has also been suggested that grounded running keeps the centre of mass low and facilitates the execution of fast turns that results from keeping one foot in contact with the ground at all times^5,21^. The birds in the current study were moving over a variable hardness snowy/icy substrate where stability will be paramount, particularly when the birds want to increase their speed, but are prevented from moving into an aerial running gait by the substrate being too compliant. By taking more frequent and shorter steps whilst keeping their centre of mass lower by selecting a grounded running gait, the birds would be able to effectively improve stability over slippery snow or ice^60^, while also increasing speed above walking range. Maintaining the centre of mass closer to the vertical plane of the contact foot improves the chance of correcting a slide on ice, something that is not an issue on the uniform rubberised substrate of a treadmill. The notion that substrate is important when considering locomotor kinematics was supported when only data for the birds grounded running over very hard snow were analysed. Very hard snow is the substrate that is as close as possible to enable a like-for-like comparison with the laboratory treadmill experiments. Examining data when the birds were moving only over very hard snow eliminated the kinematics differences found during the grounded running gait, whilst maintaining the finding of no differences between laboratory treadmill and field kinematics for walking and aerial running.

Svalbard rock ptarmigan were the first avian species for which a demonstrable decrease in the energetic cost of locomotion was found upon the switch to a high-speed aerial running gait^7^. Maximum running speed is important to the overall fitness of an animal, although it is not always the case that simply moving away the fastest is the best way to avoid predation^28^. The current study also expands the range of speed these birds can aerial run at, from the previously reported 2 ms^−1^ in the laboratory treadmill study to 2.76 ms^−1^ for birds moving in the wild, a 1.4-fold increase. Similar results have been reported for other species, for example ostriches have a 1.5-fold greater speed in the wild than on the treadmill^14,61^. These findings aren’t limited to birds, as the maximum running speed in humans and other mammals ranged from 1.7 to 2.6-fold higher when freely moving^62,63^. The treadmill underestimates top speed because these studies are principally investigating the metabolic cost of locomotion as speed increases, meaning speeds must be maintained for long enough (often 5–10 minutes) to allow stabilisation of respiratory gas measurements.

Very slow speeds (which are the most energetically expensive way to move^7^) and very high speeds (which are not aerobically sustainable for a long period of time^7^) are rarely selected by the Svalbard rock ptarmigan in the wild. The distribution of speeds in the current study indicates that the ptarmigan are making decisions linked to minimising the metabolic cost of locomotion when self-selecting speeds underpinned by the substrate they are moving over. A similar pattern has been found in other cursorial birds, where they select a narrow band of energetically optimal speeds^14,64^, a trend also found in horses^65^. The maximum attainable top speeds are likely selected as an escape strategy to move as far away as quickly as possible over a short distance rather than for sustained locomotion. Our results suggest that when conducting treadmill experiments examining animal locomotion it would be beneficial to film up to the maximum obtainable speed even if this cannot be sustained as the kinematics could then be compared to wild animals.

## Conclusion

The kinematics of locomotion are conserved across walking and aerial running gaits when Svalbard rock ptarmigan are moving under laboratory treadmill or field conditions. Walking is unaffected as moving slow negates the influence of substrate on gait while aerial running is unaffected as the birds can only use this gait over firmer ground mimicking treadmill locomotion. However, on uneven slippery ground when they want to go faster the birds must use a grounded running gait and an icy snowy substrate requires faster, shorter steps when doing this (as found for a range of animals^66^) compared to moving on a treadmill in order to maintain stability. This treadmill versus field difference disappears, however, when only field data from very hard snow conditions is considered (i.e., when differences in substrate are, as far as possible, removed). Currently the feedback mechanism the animal relies on for identifying a given substrate to move on is unknown. Two options appear possible, either that the birds rely on real time information feedback from moving over the substrate (such as substrate hardness or slipperiness) that influences gait choice and subsequent speed, or that they are able to assess substrate properties in some way, perhaps through visual inspection. However, this remains to be determined. Investigations into diurnal and seasonal time activity budgets of gait selection for the birds, likely through bio-logging (see for example e.g.^14,67,68^) would provide information of great interest towards better understanding the evolutionary significance of gait selection and the influence of substrates in the wild and contribute toward building an accurate picture of the energy budgets of wild animals and how this relates to laboratory treadmill based studies.

## Supplementary information

Marmol Guijarro_ESM

## Data Availability

All data supporting this article are provided either in the text or as part of the ESM files available through Figshare.
