# Supplementary material for: Terrestrial locomotion of the Svalbard rock ptarmigan: comparing field and laboratory treadmill studies
Source: Sci Rep. 2019 Aug 7;9:11451. doi: 10.1038/s41598-019-47989-6 (PMC6685983; doi:10.1038/s41598-019-47989-6)
Supplement: Supplementary file 1 — Marmol Guijarro_ESM [file 41598_2019_47989_MOESM1_ESM.docx]

**Supplementary Information**

Terrestrial locomotion of the Svalbard rock ptarmigan: comparing field and laboratory treadmill studies

Andres C. Marmol-Guijarro^1^, Robert L. Nudds^1^, John C. Marrin^2^, Lars P. Folkow^3^ and Jonathan R. Codd^1*^

^1^Faculty of Biology, Medicine & Health, University of Manchester, Manchester, UK. ^2^Faculty of Biological Science, University of Leeds, Leeds, UK. ^3^Department of Arctic and Marine Biology, University of Tromsø – the Arctic University of Norway, Tromsø, Norway.

**Contents**

**Figure S1…………………………………………………………………………………….2**

**Table S1……………………………………………………………………………………...3**

**Table S2……………………………………………………………………………………...4**

**Table S3……………………………………………………………………………………...5**


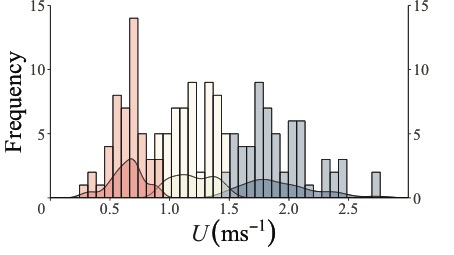


**Figure S1. Self-selected speed (*U* ms^-1^) frequency distribution across all trials.** Walking, ground running and aerial running gaits are denoted by the red, white and blue bars, respectively. The birds demonstrate a preference for walking at a relatively fast speed around 0.7ms^-1^ and to a lesser extent aerial running around 1.7-1.85ms^-1^. Generally, the birds infrequently moved at very slow (0.1-0.4ms^-1^) and very high (>2.1ms^-1^) speeds. There was no obvious preferred speed within the grounded running gait. Trials were binned into 0.07ms^-1^ speed increments across the full range. Axes show the frequency of trials.

| Term | Hand test | Estimated Ram Resistance (Swiss rammsonde) (N) | Sample of footprint |
| --- | --- | --- | --- |
|  | Object | range |  |
| Soft | Four fingers | 0­–390 | 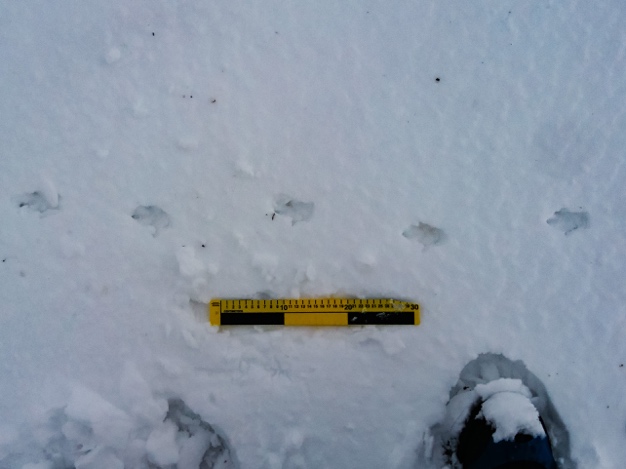 |
| Hard | Sharp pencil tip | > 390 | 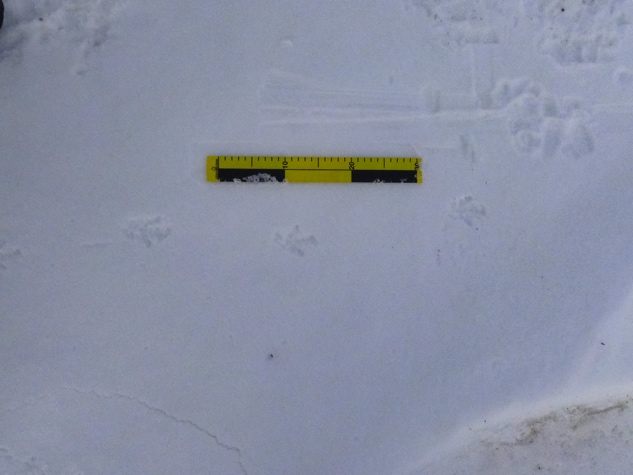 |

**Table S1**. Representative trackway footprints over snow of different hardness. Snow hardness measure are modified from [32].

| Parameter | Covariate/ factor/ interaction | Model 1 d.f. | *F* | *P* | Model 2  d.f. | *F* | *P* | Model 13  d.f. | *F* | *P* | *r*^2^ |
| --- | --- | --- | --- | --- | --- | --- | --- | --- | --- | --- | --- |
| Walking |  |  |  |  |  |  |  |  |  |  |  |
| *l*_stride_ | *U* Year Year × *U* | 1, 44  1, 44  1, 44 | 0.431  0.738  -0.429 | 0.668  0.465  0.670 | 1, 45  1, 45  * | 9.120  1.649  * | < 0.001  0.106  * | 1, 46  *  * | 9.287  *  * | < 0.001  *  * | 0.65 |
| *f*_stride_ | *U* Year Year × *U* | 1, 44  1, 44  1, 44 | -1.649  -1.960  1.651 | 0.106  0.056  0.106 | 1, 45  1, 45  * | 8.270  -1.721  * | < 0.001  0.092  * | 1, 46  *  * | 10.083  *  * | < 0.001  *  * | 0.68 |
| Grounded running |  |  |  |  |  |  |  |  |  |  |  |
| *l*_stride_ | *U* Year Year × *U* | 1, 53  1, 53  1, 53 | 1.358  1.176  1.357 | 0.180  0.245  0.180 | 1, 54  1, 54  * | 3.910  -1.176  * | < 0.001  0.245  * | 1, 55  *  * | 3.768  *  * | < 0.001  *  * | 0.19 |
| *f*_stride_ | *U* Year Year × *U* | 1, 53  1, 53  1, 53 | -1.448  -1.289  1.450 | 0.154  0.203  0.153 | 1, 54  1, 54  * | 9.318  1.017  * | < 0.001  0.314  * | 1, 55  *  * | 9.620  *  * | < 0.001  *  * | 0.62 |
| Aerial Running |  |  |  |  |  |  |  |  |  |  |  |
| *l*_stride_ | *U* Year Year × *U* | 1, 57  1, 57  1, 57 | -0.419  -0.368  0.422 | 0.676  0.715  0.675 | 1, 58  1, 58  * | 11.461  0.343  * | < 0.001  0.733  * | 1, 59  *  * | 11.543  *  * | < 0.001  *  * | 0.68 |
| *f*_stride_ | *U* Year Year × *U* | 1, 57  1, 57  1, 57 | -0.005  -0.029  0.006 | 0.996  0.977  0.995 | 1, 58  1, 58  * | 5.214  -0.158  * | < 0.001  0.875  * | 1, 59  *  * | 5.268  *  * | < 0.001 | 0.31 |

**Table S2.** Results of ANCOVAs testing for differences in *l*_stride_ and *ƒ*_stride_ between the 2017 and 2018 seasons. *l*_stride_, stride length; *ƒ*_stride,_ stride frequency. Speed (*U*) is a covariate, Year is a fixed factor and Year × *U* is the interaction term within the model, d.f. are represented as (d.f., error d.f.). The adjusted *r*^2^ values are reported for the third GLM analyses. In all cases the interaction term was not significant (Model 1) and was consequently removed from the model (Model 2). Similarly, no effect of year was found so the models were further simplified leaving only the speed term (effectively la regression analysis), which significantly affected *l*_stride_ and *ƒ*_stride_ in all gaits (Model 3).

| Gait | Parameter | model | | Slope | | Intercept | |
| --- | --- | --- | --- | --- | --- | --- | --- |
|  |  | Field | Laboratory | *z* | *p-*value | *z* | *p-*value |
| Walk |  |  |  |  |  |  |  |
|  | *l*_stride_ | 0.127 + 0.222 *U*  (*t* = 6.321, *r*^2^ = 0.63,  *n* = 25, *p* < 0.001) | 0.102 + 0.246 *U*  (*t* = 5.319, *r*^2^ = 0.96,  *n* = 3, *p* < 0.001) | -0.426 | 0.667 | 0.705 | 0.478 |
|  | *ƒ*_stride_ | 1.258 + 1.744 *U*  (*t* = 5.208, *r*^2^ = 0.54,  *n* = 25, *p* < 0.001) | 1.043 + 2.130  (*t* = 36.28, *r*^2^ = 0.99,  *n* = 3, *p* < 0.001) | -1.137 | 0.271 | 0.941 | 0.347 |
|  | *t*_stance_ | -0.666 – 0.619 log_10_ *U*  (*t* = -6.257, *r*^2^ = 0.80,  *n* = 12, *p* < 0.001) | -0.646 – 0.623 log_10_ *U*  (*t* = -7.368, *r*^2^ = 0.98,  *n* = 3, *p* < 0.086) | 0.040 | 0.992 | -0.642 | 0.522 |
|  | *t*_swing_ | -0.797 + 0.200 log_10_ *U*  (*t* = 1.507, *r*^2^ = 0.19,  *n* = 12, *p* = 0.163) | -0.838 – 0.012 log_10_ *U*  (*t* = -0.20, *r*^2^ = 0.03,  *n* = 3, *p* = 0.88) | 1.447 | 0.147 | 0.998 | 0.317 |
|  | DF | 0.871 – 0.330 *U*  (*t* = -4.746, *r*^2^ = 0.69,  *n* = 12, *p* < 0.001) | 0.858 – 0.281 *U*  (*t* = -6.72, *r*^2^ = 0.98,  *n* = 3, *p* = 0.094) | -0.589 | 0.555 | 0.261 | 0.795 |
| Grounded running |  |  |  |  |  |  |  |
|  | *l*_stride_ | 0.211 + 0.122 *U*  (*t* = 4.353, *r*^2^ = 0.47,  *n* = 23, *p* < 0.001) | 0.194 + 0.158 *U*  (*t* = 7.934, *r*^2^ = 0.95,  *n* = 5, *p* < 0.01) | -0.888 | 0.373 | – | – |
|  | *ƒ*_stride_ | 1.400 + 1.624 *U*  (*t* = 6.737, *r*^2^ = 0.68,  *n* = 23, *p* < 0.001) | 1.621 + 1.258 *U*  (*t* = 5.728, *r*^2^ = 0.92,  *n* = 5, *p* < 0.05) | 1.203 | 0.230 | – | – |
|  | *t*_stance_ | -0.715– 0.896 log_10_ *U*  (*t* = -5.738, *r*^2^ = 0.75,  *n* = 13, *p* < 0.001) | -0.656 – 0.955 log_10_ *U*  (*t* = -44.49, *r*^2^ = 0.99,  *n* = 5, *p* < 0.001) | 0.335 | 0.734 | – | – |
|  | *t*_swing_ | -0.877 – 0.021 log_10_ *U*  (*t* = 0.123, *r*^2^ = 0.001,  *n* = 13, *p* < 0.01) | -0.796 + 0.00 log_10_ *U*  (*t* = 0, *r*^2^ = 0.51,  *n* = 5, *p* = 0.167) | -0.117 | 0.904 | – | – |
|  | DF | 0.739 – 0.157 *U*  (*t* = -3.442, *r*^2^ = 0.52,  *n* = 13, *p* < 0.001) | 0.734 – 0.164 *U*  (*t* = -18.18, *r*^2^ = 0.99,  *n* = 5, *p* < 0.001) | 0.140 | 0.889 | – | – |
| Aerial Running |  |  |  |  |  |  |  |
|  | *l*_stride_ | 0.207 + 0.126 *U*  (*t* = 8.034, *r*^2^ = 0.75,  *n* = 24, *p* <0.001) | 0.247 + 0.131 *U*  (*t* = 2.484, *r*^2^ = 0.75,  *n* = 4, *p* = 0.131) | -0.104 | 0.920 | – | – |
|  | *ƒ*_stride_ | 2.384 + 0.981 *U*  (*t* = 6.354, *r*^2^ = 0.65,  *n* = 24, *p* < 0.001) | 1.788 + 1.078 *U*  (*t* = 3.12, *r*^2^ = 0.82,  *n* = 4, *p* = 0.089) | -0.256 | 0.795 | – | – |
|  | *t*_stance_ | -0.770 - 0.726 log_10_ *U*  (*t* = -4.403, *r*^2^ = 0.62,  *n* = 14, *p* < 0.01) | -0.698 – 0.723 log_10_ *U*  (*t* = -3.32, *r*^2^ = 0.85,  *n* = 4, *p* = 0.08) | -0.012 | 0.992 | – | – |
|  | *t*_swing_ | -0.797 -0.380 log_10_ *U*  (*t* = -2.839, *r*^2^ = 0.4,  *n* = 14, *p* = 0.24) | -0.722 – 0.311 log_10_ *U*  (*t* = -1.71, *r*^2^ = 0.59,  *n* = 4, *p* = 0.23) | -1.684 | 0.093 | – | – |
|  | DF | 0.539 -0.041 *U*  (*t* = -1.449, *r*^2^ = 0.15,  *n* = 14, *p* = 0.045) | 0.621 – 0.095 *U*  (*t* = -1.992, *r*^2^ = 0.67,  *n* = 4, *p* < 0.185) | 0.967 | 0.332 | – | – |

**Table S3.** Results of the linear regressions of each kinematics parameter against *U* for each gait and the *z*-test comparisons of the slope and intercept coefficients for laboratory data and field data collected from birds moving over hard snow. The lines of best fit are also given. Only the intercepts for the walking gaits were compared, because comparison for grounded running and aerial running would require extrapolating the lines of best fit too far beyond the data range, rendering their estimates unreliable.
